# Pomegranate peel extract protects against the development of diabetic cardiomyopathy in rats by inhibiting pyroptosis and downregulating LncRNA-MALAT1

**DOI:** 10.3389/fphar.2023.1166653

**Published:** 2023-03-28

**Authors:** Mariam Ali Abo-Saif, Amany E. Ragab, Amera O. Ibrahim, Othman F. Abdelzaher, Ahmed B. M. Mehanyd, Maha Saber-Ayad, Ola A. El-Feky

**Affiliations:** ^1^ Department of Biochemistry, Faculty of Pharmacy, Tanta University, Tanta, Egypt; ^2^ Department of Pharmacognosy, Faculty of Pharmacy, Tanta University, Tanta, Egypt; ^3^ Zoology Department, Faculty of Science, Al-Azhar University, Cairo, Egypt; ^4^ Department of Clinical Sciences, College of Medicine and Research Institute for Medical and Health Sciences, University of Sharjah, Sharjah, United Arab Emirates; ^5^ Department of Pharmacology, College of Medicine, Cairo University, Giza, Egypt

**Keywords:** long non-coding RNA-metastasis-associated lung adenocarcinoma transcript-1, NLRP3, caspase-1, IL1β, TGF-β, pomegranate peel extract

## Abstract

**Background:** Pyroptosis is an inflammatory programmed cell death accompanied by activation of inflammasomes and maturation of pro-inflammatory cytokines interleukin-1β (IL-1β) and IL-18. Pyroptosis is closely linked to the development of diabetic cardiomyopathy (DC). Pomegranate peel extract (PPE) exhibits a cardioprotective effect due to its antioxidant and anti-inflammatory properties. This study aimed to investigate the underlying mechanisms of the protective effect of PPE on the myocardium in a rat model of DC and determine the underlying molecular mechanism.

**Methods:** Type 1 diabetes (T1DM) was induced in rats by intraperitoneal injection of streptozotocin. The rats in the treated groups received (150 mg/kg) PPE orally and daily for 8 weeks. The effects on the survival rate, lipid profile, serum cardiac troponin-1, lipid peroxidation, and tissue fibrosis were assessed. Additionally, the expression of pyroptosis-related genes (NLRP3 and caspase-1) and lncRNA-MALAT1 in the heart tissue was determined. The PPE was analyzed using UPLC-MS/MS and NMR for characterizing the phytochemical content.

**Results:** Prophylactic treatment with PPE significantly ameliorated cardiac hypertrophy in the diabetic rats and increased the survival rate. Moreover, prophylactic treatment with PPE in the diabetic rats significantly improved the lipid profile, decreased serum cardiac troponin-1, and decreased lipid peroxidation in the myocardial tissue. Histopathological examination of the cardiac tissues showed a marked reduction in fibrosis (decrease in collagen volume and number of TGF-β-positive cells) and preservation of normal myocardial structures in the diabetic rats treated with PPE. There was a significant decrease in the expression of pyroptosis-related genes (NLRP3 and caspase-1) and lncRNA-MALAT1 in the heart tissue of the diabetic rats treated with PPE. In addition, the concentration of IL-1β and caspase-1 significantly decreased in the heart tissue of the same group. The protective effect of PPE on diabetic cardiomyopathy could be due to the inhibition of pyroptosis and downregulation of lncRNA-MALAT1. The phytochemical analysis of the PPE indicated that the major compounds were hexahydroxydiphenic acid glucoside, caffeoylquinic acid, gluconic acid, citric acid, gallic acid, and punicalagin.

**Conclusion:** PPE exhibited a cardioprotective potential in diabetic rats due to its unique antioxidant, anti-inflammatory, and antifibrotic properties and its ability to improve the lipid profile. The protective effect of PPE on DC could be due to the inhibition of the NLRP3/caspase-1/IL-1β signaling pathway and downregulation of lncRNA-MALAT1. PPE could be a promising therapy to protect against the development of DC, but further clinical studies are recommended.

## 1 Introduction

Diabetic patients with poor glycemic control have a high probability of developing diabetic cardiomyopathy (DC) and heart failure. The pathophysiology of DC is linked to impaired glucose metabolism in diabetic myocardium caused by reduced insulin signaling and the heart’s dependence on fatty acid oxidation for energy (Yan et al., 2020). Altered glucose and lipid metabolism in diabetic cardiomyocytes increases mitochondrial production of nitric oxide radicals and stimulates multiple inflammatory pathways. Prominent oxidative stress, chronic inflammation, and increased production of advanced glycation end-products (AGEs) lead to myocardial tissue damage with pathological remodeling of the cardiac tissue and fibrosis (Prandi et al., 2022).

Pyroptosis is an inflammatory programmed cell death activated in response to microbial infection, cellular damage, or metabolic imbalances (Xue et al., 2019). Pyroptosis is characterized by the formation of the nucleotide-binding domain (NOD)-like receptor protein 3 (NLRP3) inflammasome complex that is composed of NLRP3, apoptosis-associated speck-like protein (ASC), and pro-caspase-1. Oligomerization of NLRP3 inflammasome mediates the activation of caspase-1 and the release of pro-inflammatory cytokines IL-1β and IL-18 (Zheng and Li, 2020).

Previous studies have shown that pyroptosis activation is associated with the development of DC. Reactive oxygen species (ROS) and hyperglycemia activate NLRP3 oligomerization and the associated inflammatory programmed cell death, which can progress to DC (Lu et al., 2022). Moreover, Meng et al. (2022) reported that the downregulation of NLRP3 inflammasome restores myocardial function in DC models.

Long non-coding RNAs (lncRNAs) are a group of RNA transcripts that comprise more than 200 nucleotides, but they do not have the ability to encode protein. LncRNAs are involved in different pathological mechanisms in various diseases (Khorkova et al., 2015). Metastasis-associated lung adenocarcinoma transcript-1 (MALAT1) is an lncRNA which is essential in the regulation of different biological pathways and has been reported to be involved in the pathogenesis of DC (Zhang et al., 2016; Abdulle et al., 2019). Recently, Shi et al. (2021) reported that lncRNA-MALAT1 is upregulated in DC and that knockdown of MALAT1 protects against the development of DC by affecting the NLRP3 formation.

So far, there has been no specific treatment addressing the pathogenesis of DC. The cardioprotective strategies for DC prevention include the use of antioxidants, antifibrotic agents, and anti-inflammatory agents (Lorenzo-Almorós et al., 2022). Botanicals from plants are an invaluable source of biological effects. They include many classes of compounds, of which polyphenolic compounds, triterpenes, saponins, alkaloids, and glycosides are common. Polyphenolic compounds are strong antioxidants common in fruits and vegetables. Pomegranate fruit (*Punica granatum* L., family Lythraceae) is rich in polyphenolic compounds with strong antioxidative and anti-inflammatory properties (Ismail et al., 2012). Moreover, punicalagin (an active constituent extracted from pomegranate peel) can induce pyroptosis in a collagen-induced arthritis model (El-Mansi and Al-Kahtani, 2019; Ge et al., 2022).

Regeneration of pancreatic ß cells may be enhanced by the pomegranate peel aqueous extract (Punasiya et al., 2010). Previous studies showed that pomegranate peel extract (PPE) has antidiabetic and cardiovascular protection properties *in vitro* (Arun et al., 2017), and it also has a cardioprotective effect in a diabetic rat model (Albarakti, 2016). In addition, the study by El-Mansi and Al-Kahtani (2019) demonstrated that PPE showed a cardioprotective effect on diabetic mother rats and their neonates, and the study recommended the use of PPE as a promising treatment, which protects against the secondary myocardial complications of diabetes. Moreover, a recent study by Dab et al. (2022) reported that PPE improves the cardiac extracellular matrix remodeling and decreases fibrosis in diabetic rats. Interestingly, PPE may exhibit a liver protective effect and reduce histological and functional changes in a rat model of diabetes (Faddladdeen and Ojaimi, 2019).

This study aimed to investigate the underlying mechanisms of the potential protective effect of PPE against DC. Intriguingly, we demonstrated, for the first time that PPE interferes with pyroptosis by inhibiting the NLRP3/caspase-1/IL1β signaling pathway through downregulating lncRNA-MALAT1.

## 2 Materials and methods

### 2.1 Plant material

Pomegranate (*Punica granatum* L. var. Edkawy) fruits were obtained from a private farm in Edku city, Al-Beheira Governorate, and verified by the Agricultural Research Center in Cairo. The peel of the fruits was washed with water and air dried. A voucher sample (PD-201-AR) was kept at the Department of Pharmacognosy, Faculty of Pharmacy, Tanta University. After complete drying, the peel was kept in an oven at 40°C for 10 h to ensure the removal of all water content, and then it was ground into fine powder. The powdered material (800 g) was extracted by 70% methanol (25 mL solvent per 1 g powder) using a magnetic stirrer overnight. The extract was filtered, and the process was repeated for 3 days to ensure exhaustion. The combined solvent filtrates were evaporated under vacuum to leave a reddish-brown residue (yield, 15% w/w).

### 2.2 Phytochemical characterization of the extract

The PPE was analyzed using UPLC-PDA-MS/MS following a previously reported methodology (Ragab et al., 2022; Assar et al., 2021a; Assar et al., 2021b). A Nexera-i LC-2040 (Shimadzu, Kyoto, Japan) system connected to a UPLC C_18_ column (Shim-pack Velox, 2.1 × 50 mm; 2.7 μm particle) was used. The mobile-phase gradient and the preparation of the sample were as reported (Assar et al., 2021a; Assar et al., 2021b; Ragab et al., 2022). The mobile phase gradient (solvent A: water containing 0.1% formic acid; solvent B: acetonitrile) at a flow rate of 0.2 mL/min: 0–2 min: 10% B; 2–5: linear gradient to 30% B; 5–15 min: linear gradient to 70% B; 15–22 min: linear gradient to 90% B; 22–25 min: linear gradient to 95% B; 25–26 min: linear gradient to 100% B; 26–29 min: isocratic 100% B; 29–30 min: linear gradient to 10% B was used. A PDA detector (LC-2030/2040) and a triple quadrupole mass spectrometer (LC-MS 8045) equipped with an electrospray ionization (ESI) source in a negative mode (Shimadzu, Kyoto, Japan) were used for detecting the compounds using the reported settings (Assar et al., 2021a; Assar et al., 2021b; Ragab et al., 2022).

A Bruker Avance 400 spectrophotometer at 400 MHz was used for the ^1^H NMR analysis of the PPE. DMSO-*d*_*6*_ was used as a solvent. The method development parameters were the same as those published in our recent work on pomegranate extract from the Manfaloty variety (Ragab et al., 2022).

### 2.3 Biological evaluation

#### 2.3.1 Animal model and experimental design

This study was carried out based on the Guidelines for the Care and Use of Laboratory Animals and approved by the Research Ethical Committee, Faculty of Pharmacy, Tanta University, Egypt (ethical approval code: TP/RE/06/22P-0016). Forty-eight male Wistar rats, aged 6 weeks, and weighing about 120–150 g, were purchased from the National Research Center (Cairo, Egypt). The rats were fed pellet chow (El-Nasr Chemical^®^, Egypt) and allowed free access to diet and water. The animals were housed under identical environmental conditions and maintained for 2 weeks for acclimatization (Torika et al., 2018).

After the acclimatization, the animals were randomly divided into four groups (*n* = 15): negative control group (normal rat), positive control group (diabetic rats), diabetic treated group (diabetes rats treated with PPE), and normal treated group (normal rats treated with PPE). Type 1 diabetes mellitus (T1DM) was induced by a single ip dose of 60 mg/kg streptozotocin (Sigma Aldrich, St. Louis, United States) dissolved in 0.1 M sodium citrate buffer. Type 1 diabetes developed 72 h after streptozotocin injection. Rats in the negative control group received a single ip dose of the vehicle (0.1 M sodium citrate buffer 0.25 mL/kg).

Only those rats with blood glucose concentrations greater than 16.7 mmol/L (300 mg/dL) were considered diabetic and were chosen to complete the study. Blood glucose was determined using a blood glucose meter Accu-Chek Performa (Roche Diagnostics, Indianapolis, United States) by tail vein puncture (Babu and Srinivasan, 1997; Riazi et al., 2006).

Rats in the diabetic treated group and non-diabetic treated group were given 1 mL of the PPE at a dose of 150 mg/kg orally and daily for eight consecutive weeks, while rats in the negative and positive control groups were given 1 mL of the vehicle orally and daily for eight consecutive weeks model (El-Mansi and Al-Kahtani, 2019; Mayyas et al., 2021).

At the end of our experiment, the total body weight of the rats was measured, and random blood glucose levels were determined using a blood glucose meter. The rats were fasted for 16 h and sacrificed. Blood was collected by cardiac puncture, and the serum was separated for determination of lipid profile and serum cardiac troponin I (cTn1). The heart was removed and washed with cold saline (pH = 7.4), and the weight of the heart was determined. The heart was cut into two portions; one portion was fixed in 10% formaldehyde for histopathological evaluation and immunohistochemical staining of transforming growth factor beta (TGF-β), while the other portion was kept at −80°C for biochemical estimations of malondialdehyde (MDA), enzyme-linked immunosorbent assay (ELISA) of interleukin 1 beta (IL-1β) and caspase-1, and quantitative reverse transcriptase-polymerase chain reaction (qRT-PCR) for gene expression of pyroptosis markers (NLRP3 and caspase-1) and lncRNA-MALAT1.

#### 2.3.2 Determination of fasting serum lipid profile

A cholesterol assay kit—HDL and LDL/VLDL (Abcam, Cambridge, United Kingdom)—was used to determine total cholesterol (T-Chol), high-density lipoprotein cholesterol (HDLc), and low-density lipoprotein cholesterol/very-low-density lipoprotein cholesterol (LDLc/VLDLc) in the serum of the studied rat groups according to the manufacturer’s instructions. The kit quantifies T-Chol by an enzymatic colorimetric method and includes a simple technique to separate HDLc and LDLc/VLDLc for their determination. The color intensity of the reaction product is measured at 570 nm, and it is directly proportional to cholesterol levels.

A low-density lipoprotein cholesterol colorimetric assay kit (Elabscience, Wuhan, China) was used to determine LDLc in the serum of the rat groups according to the manufacturer’s instructions. The kit quantifies LDLc using an enzymatic colorimetric method, and the color intensity of the reaction product is measured at 546 nm using a microplate reader (Thermo Fisher Scientific, Waltham, MA, United States). The determined value of LDLc was used to obtain the VLDLc level by subtracting the LDLc value from the non-HDLc (LDLc/VLDLc) value.

A triglyceride (TG) colorimetric assay kit (Elabscience, Wuhan, China) was used to determine TG in the serum of the rat groups according to the manufacturer’s instructions. The color intensity of the generated quinones is directly proportional to the TG content. The absorbance was measured at 240 nm using a spectrophotometer (Analytik Jena, Jena, Germany) (Konda et al., 2017).

#### 2.3.3 ELISA for the determination of cardiac troponin I, IL1-β, and caspase-1

A rat cardiac troponin I ELISA kit (Abcam, Cambridge, United Kingdom) was used for the determination of cardiac troponin I (a marker of myocardial injury) in the serum of the rat groups according to the manufacturer’s protocol (Jin et al., 2021). In addition, a rat IL1-β ELISA kit (Abcam, Cambridge, United Kingdom) and a rat caspase-1 ELISA kit (MyBioSource, California, United States) were used for the determination of IL1-β (pro-inflammatory cytokine released as a result of NLRP3 activation) and caspase-1 (IL-1 converting enzyme), respectively, in the heart tissues, according to the manufacturer’s protocol. The colored intensity of the final product was measured at 450 nm using a microplate reader (Thermo Fisher Scientific, Waltham, MA, United States).

#### 2.3.4 Determination of oxidative stress in myocardial tissue

The degree of myocardial lipid peroxidation was determined by measuring the level of MDA using an MDA assay kit (Biodiagnostic, Cairo, Egypt). According to the manufacturer’s protocol, a pink color is produced by the reaction of thiobarbituric acid with MDA. The absorbance was measured at 534 nm using a spectrophotometer (Analytik Jena^®^, Jena, Germany) (Ohkawa et al., 1979).

#### 2.3.5 RNA extraction and qRT-PCR for lncRNA-MALAT1, NLRP3, and caspase-1

Total RNA from the cardiac tissue was extracted using an miRNeasy^®^ Mini Kit (Qiagen Co., Hilden, Germany) and reverse-transcribed to the cDNA with the two-step RT-PCR kit (Qiagen Co., Hilden, Germany). The PCR was performed with QuantiTect SYBR Green I PCR (Qiagen Co., Hilden, Germany) by denaturing at 95°C for 10 s, annealing at 60°C for 15 s and extending at 72°C for 25 s. The primers were obtained from Willowfort Co. (Birmingham, England), and the primer sequences were as follows: MALAT1 (NR_144568.1) forward (5′-AAA​GCA​AGG​TCT​CCC​CAC​AAG-3′) and reverse: (5′-GGT​CTG​TGC​TAG​ATC​AAA​AGG​CA-3′) (Wu et al., 2020), NLRP3 (NM_001191642.1) forward (5′-AGC​CTC​AGG​GCA​CCA​AA-3′) and reverse (5′-GGT​ATG​GCG​TGG​CAA​GAG​TC-3′), caspase-1 (NM_012762.3) forward (5′-ATG​GAT​TGC​TGG​ATG​AAC T-3′) and reverse (5′-GAT​AAC​CTT​GGG​CTT​GTC​TT- 3′), and GAPDH (NM_017008.4) forward (5′-TCC​ATG​ACA​ACT​TTG​GCA​TC-3′) and reverse (5′-CAT​GTC​AGA​TCC​ACC​ACG​GA-3′) (Du et al., 2018). The gene expression was calculated using the 2^−ΔΔCT^ method (Heid et al., 1996).

#### 2.3.6 Histopathological examination

At the end of our experiment, the heart sections of four rats were selected randomly from each group. The heart tissues were fixed in 10% neutral formalin for 12 h. Each sample was then soaked in paraffin, and sections (5-μm thick) were cut and stained with hematoxylin-eosin (H&E). The sections were evaluated using an optical microscope (CX43; Olympus, Tokyo, Japan) for the detection of histopathological abnormality (Saber et al., 2020).

#### 2.3.7 Immunohistochemical staining of TGF-β as a marker for fibrosis

Immunohistochemical demonstration of TGF-β in paraffin-embedded heart sections was performed using the TGF-β kit (Sigma Aldrich, St. Louis, United States). Cardiac tissues were fixed with 10% neutral formaldehyde. Tissue sections were cut (5-μm thick) and deparaffinized. The endogenous peroxidase was quenched with two drops of 3% H_2_O_2_ for 5 min. Then, the sections were treated with a blocking reagent for 10 min. The primary antibody (rabbit anti-cyclooxygenase-2 in buffered saline) or the negative control was added and incubated for 1 hour. Then, the secondary biotinylated antibody (goat anti-rabbit IgG in buffered saline) was added and incubated for 20 min. Peroxidase was applied and incubated for another 20 min. A substrate reagent and aminoethylcarbazole in *N*, *N*-dimethylformamide chromogen were applied and incubated for 10 min. Mayer’s hematoxylin was used for immunohistochemistry (IHC) counterstain. Six typical fields were selected randomly from each slice for observation under an optical microscope (CX43; Olympus, Tokyo, Japan). The cytoplasm of the positive cells was red-rose to brownish-red (Kuo et al., 2005).

#### 2.3.8 Masson’s trichrome stain for determination of collagen in the rat myocardium

For determining the collagen content of the rat myocardium, Masson’s trichrome staining kit (Beijing Solarbio Science & Technology, Beijing, China) was used to stain the rat myocardium. The method of staining was modified from the method used by Ukong et al. (2008). The slides stained with Masson’s trichrome stain were examined using a polarized light microscope (Leica, Wetzlar**,** Germany), and the intensity of the blue color was determined (representing the collagen density) using a software image analyzer.

### 2.4 Statistical analysis

The data are expressed as the mean ± SD, and SPSS version 22 software was used for the statistical analysis. The statistical comparison between groups was analyzed by one-way analysis of variance (ANOVA), followed by *post-hoc* Fisher’s least significant difference (LSD). *p* < 0.05 was considered statistically significant.

## 3 Results

### 3.1 Phytochemical analysis of the PPE

**FIGURE 1:**
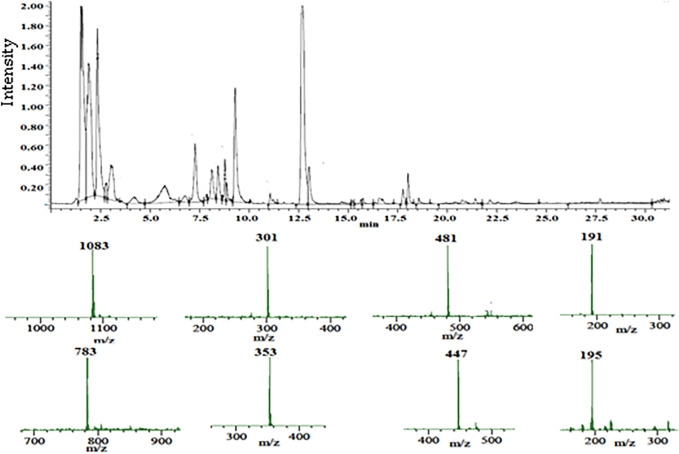
Total ion chromatogram of the UPLC-PDA-MS/MS analysis of the pomegranate peel extract PPE (top), and the mass spectra of the major constituents (bottom).

In a negative ion mode, the UPLC-PDA-MS/MS analysis of the PPE detected 28 peaks (Figure 1). The compounds were identified (Table 1) by comparing the retention times, mass, and MS/MS data to the published literature for pomegranate (Abid et al., 2017; Yisimayili et al., 2019; García et al., 2021; Ragab et al., 2022). According to the area under the peaks, the major compounds identified are gluconic acid (18.39%), caffeoylquinic acid (14.0%), citric acid (19.53%), punicalagin (7.84%), delphinidin-3-*O*-glucoside (1.80%), ellagic acid-*O*-pentoside (0.68%), ellagic acid (18.26%), and hexahydroxydiphenoyl glucoside (HHDPG, 21.54%). The latter compound is the prevalent phenolic compound in the extract (21.54%) based on the area under the peak.

**TABLE 1:** UPLC-PDA-MS/MS analysis results of pomegranate peel extract (PPE).

No.	Rt min	[M-H]^-^ (*m/z*)	MS^2^ ions (*m/z*)	Identification
1	1.50	195	177, 159, 129, 99, and 75	Gluconic acid
2	1.89	353	261, 219, 191, 173, 149, and 113	Caffeoylquinic acid
3	2.34	191	173 and 111	Citric acid
4	2.80	205	205	Unknown
5	3.02	205	205	Unknown
6	4.23	783	481, 301, and 275	Pedunculagin I
7	5.72	541*	541 and 302	Punicalagin
	7.71	1083	1083, 781, 603, 601, 575, 541, and 302	
8	6.73	783	765, 481, 301, and 275	Pedunculagin II
9	7.84	801	649, 348, 347, and 301	Punigluconin
10	8.13	463	302	Delphinidin-3-*O*-glucoside
11	8.42	469	425, 301, 169, and 125	Valoneic acid dilactone
12	8.86	447	345, 259, 219, 160, and 113	Cyanidin-3-*O*-glucoside
14	9.28	301	301, 229, and 185	Ellagic acid
15	12.50–13.50	481	301	HHDPG
16	15.21	213	ND	Unknown
17	15.64	499	455 and 437	Carboxy ursolic acid
18	15.75	485	455, 422, and 365	Methoxy ursolic acid
19	16.56	487	455, 469, 467, 421, 392, and 375	Dihydroxy ursolic acid
21	17.76	485	455, 422, and 365	Methoxy ursolic acid isomer
22	18.02	487	455, 469, 467, 421, 392, and 375	Dihydroxy ursolic acid isomer
24	21.41	471	423, 407, 405, and 393	Maslinic acid
27	27.71	339	339	Behenic acid
28	31.38	487	487	Asiatic acid

ND, not detected; *, [M-2H]; Rt, retention time.

**FIGURE 2:**
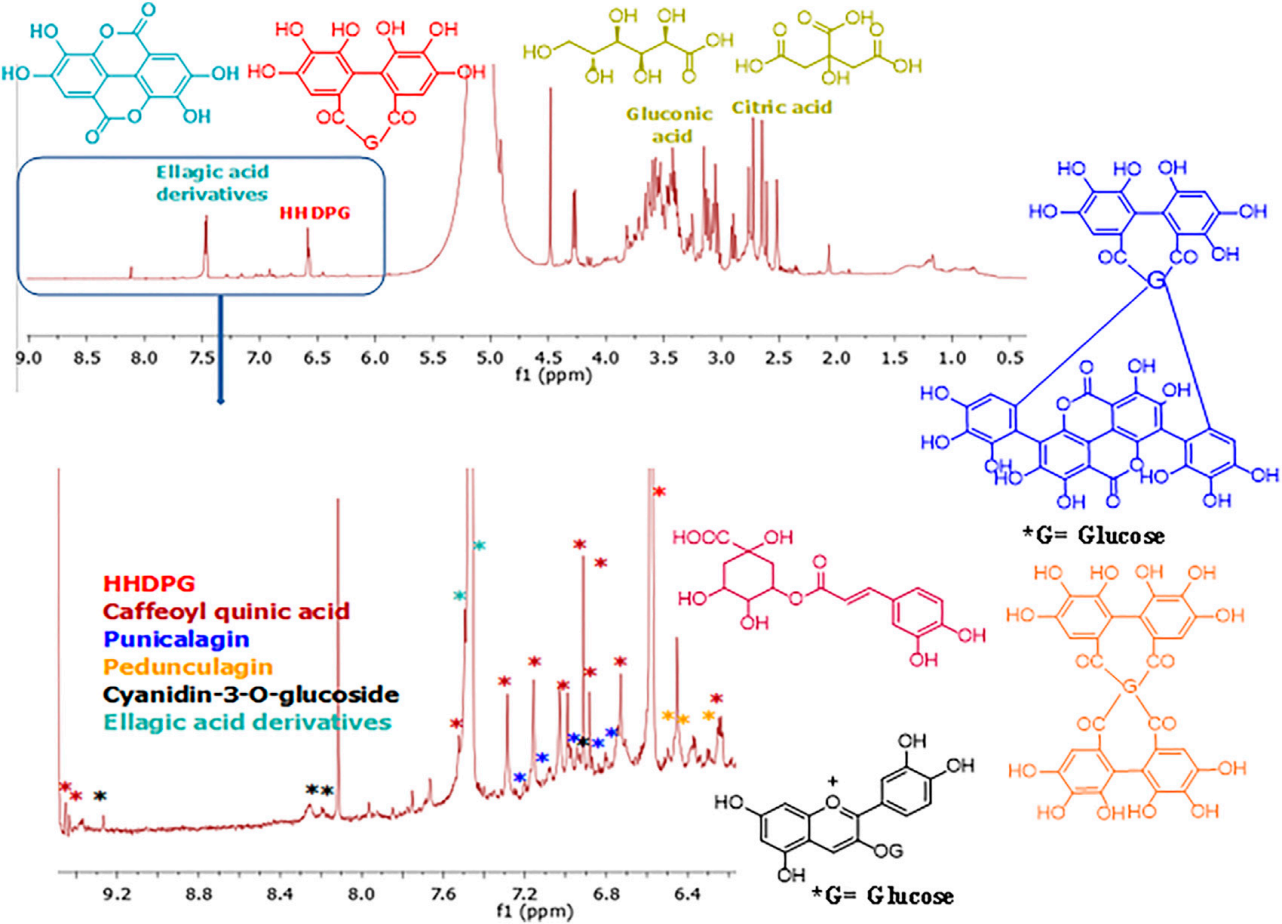
^1^H NMR fingerprint of the pomegranate peel extract PPE (Top) and expanded aromatic protons region (6.30–9.50 ppm) (bottom). The structures of the detected compounds are shown. Red: HHDPG, dark red: caffeoyl quinic acid, dark blue: punicalagin, orange: pedunculagin, black: cyanidin-3-O-glucoside, light blue: ellagic acid derivatives.

The ^1^H NMR analysis of the PPE (Figure 2) indicated that the characteristic signals for HHDPG, ellagic acid, gluconic acid, and citric acid were predominant compared to the published literature (Guy, 1998; Bajko et al., 2016; Wan et al., 2017; Ahmed et al., 2019; Gabr et al., 2019; Tian et al., 2019; Hammoud Mahdi et al., 2020; Hasanpour et al., 2020). The signals at δ_H_ 6.51 and 6.52 were assigned to H-6′ and H-6″ in HHDP moiety (Gabr et al., 2019), respectively, while the signals at at δ_H_ 7.42 and 7.46 were assigned to H-5 and H-5′ in ellagic acid derivatives (Ahmed et al., 2019), respectively. The signals at δ_H_ 2.64 and 2.73 were ascribed to the symmetrical methylene diastereotopic protons of citric acid (Guy, 1998). The signals at δ_H_ 3.40–3.75 were ascribed to the protons of gluconic acid (Hammoud Mahdi et al., 2020). The signals at δ_H_ 6.30, 6.77, 6.92, 7.04, 7.53, 9.43, and 9.53 are characteristic of caffeoylquinic acids (Bajko et al., 2016; Wan et al., 2017). Signals for cyanidin-3-*O*-glucoside appeared at δ_H_ 6.44, 6.93, 8.21, 8.26, and 9.25, which are consistent with the literature (Tian et al., 2019). Signals at δ_H_ 6.72, 6.88, 6.90, 6.97, 7, 05, 7.12, 7.21, and 7.26 were attributed to the aromatic protons of α and β punicalagin as compared to the literature (Hasanpour et al., 2020). Signals at δ_H_ 6.30, 6.31, 6.50, 6.54, 6.59, 6.60, 6.63, and 6.64 were consistent with pedunculagin (Khanbabaee and Großer, 2003). Other compounds detected by UPLC-PDA-MS were below the detection limit by NMR under the used concentration and conditions.

### 3.2 Biological evaluation

#### 3.2.1 Effect on cardiac hypertrophy in the diabetic rats and the survival rate

The overall survival rate of rats was 76.66% (46/60). In the negative control group, positive control group, diabetic treated group, and only treated group, the numbers of survival were 14, 8, 11, and 13, respectively, and the survival rates of the rats were 93.33%, 53.33%, 73.33%, and 86.67%, respectively.

**FIGURE 3:**
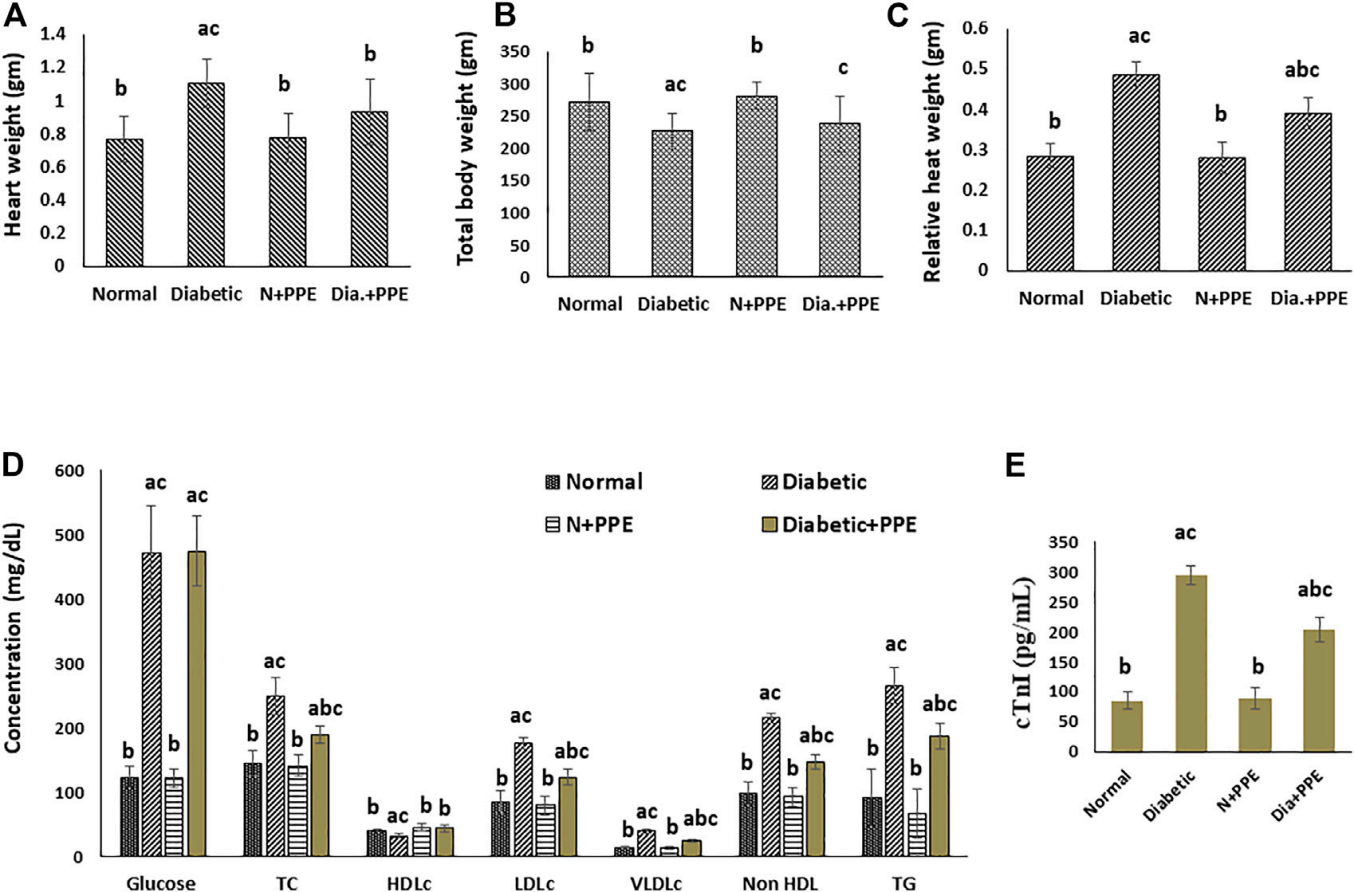
Effect of the pomegranate peel extract treatment on the studied rat groups **(A)** heart weight, **(B)** total body weight, **(C)** relative heart weight, **(D)** random blood glucose and fasting serum lipids, and **(E)** serum cardiac troponin-I concentration. Data are presented as mean ± SD; n = 7. cTn1: cardiac troponin I, Normal: normal rats (negative control group), Diabetic: non-treated diabetic rats (positive control group), N+PPE: normal rats treated with the pomegranate peel extract, Dia+PPE: diabetic rats treated with the pomegranate peel extract. HDLc: high density lipoprotein cholesterol, LDLc: low density lipoprotein cholesterol, Non HDL: non high density lipoprotein, TC: total cholesterol, TG: triglyceride, VLDLc: very low density lipoprotein cholesterol.

At the end of our experiment, the total body weight of the rats was determined. The weight of the heart was determined after animal sacrifice, and the relative heart weight was calculated. The present study showed that the relative heart weight of the diabetic rats in the positive control group (0.488 ± 0.030 g) was significantly higher than the relative heart weight of the normal rats in the negative control group (0.2851 ± 0.033 g) (*p* < 0.001). Prophylactic treatment with the PPE in the diabetic rats significantly decreased the relative heart weight (0.391 ± 0.043 g) compared to the untreated diabetic rats in the positive control group (*p* < 0.001). In addition, the relative heart weight of the non-diabetic normal rats treated with the PPE showed no significant difference from the relative heart weight of the untreated normal rats in the negative control group (Figures 3A–C).

#### 3.2.2 Effect on fasting serum lipid profile and random blood glucose concentration

Fasting serum profile and random blood glucose of the studied rat groups were determined at the end of our experiment (Figure 3D). Prophylactic treatment with the PPE significantly decreased (*p* < 0.001) total cholesterol (188.50 ± 13.16 mg/dL), low-density lipoprotein cholesterol (122.75 ± 11.79 mg/dL), very-low-density lipoprotein cholesterol (23.88 ± 2.03 mg/dL), non-high-density lipoprotein cholesterol (146.75 ± 11.61 mg/dL), triglycerides (186.43 ± 20.55 mg/dL), and the total cholesterol/HDLc risk ratio (4.38 ± 0.56) levels in the serum of the diabetic rats compared to their levels in the serum of the untreated diabetic rats in the positive control group 248.50 ± 28.69 mg/dL, 175.88 ± 7.93 mg/dL, 39.63 ± 3.07 mg/dL, 215.50 ± 7.65 mg/dL, 265.00 ± 28.02 mg/dL, and 7.64 ± 0.56, respectively. Moreover, the HDLc levels increased significantly in the serum of the diabetic rats treated with the PPE (43.75 ± 4.77 mg/dL) compared to the untreated diabetic rats in the positive control group (32.00 ± 3.07 mg/dL) (*p* < 0.001). The random blood glucose concentrations were significantly higher in the diabetic rats in the positive control group (472.38 ± 72.18 mg/dL) than in the normal rats in the negative control group (123.43 ± 15.98 mg/dL) (*p* < 0.001), but the blood glucose concentrations of the diabetic rats treated with the PPE showed no significant difference from blood glucose concentrations of the untreated diabetic rats in the positive control group.

#### 3.2.3 Effect on serum cardiac troponin 1

Cardiac troponin I concentration in the serum of the studied rat groups was determined as a marker of myocardial injury. The concentration of serum cTn1 was significantly higher in the diabetic rats of the positive control group (294.13 ± 16.09 pg/mL) than in the non-diabetic rats in the negative control group (85.00 ± 14.64 pg/mL) (*p* < 0.001). Moreover, the prophylactic treatment of the diabetic rats with the PPE significantly decreased serum cTn1 levels (203.63 ± 20.98 pg/mL) compared to the cTn1 concentration in the serum of the untreated diabetic rats in the positive control group (*p* < 0.001). The concentration of cTn1 in the serum of the normal rats treated with the PPE (88.43 ± 18.42 pg/mL) showed no significant difference from the concentration of cTn1 in the serum of rats in the negative control group (Figure 3E).

#### 3.2.4 Effect on oxidative stress marker in the myocardial tissue

**FIGURE 4:**
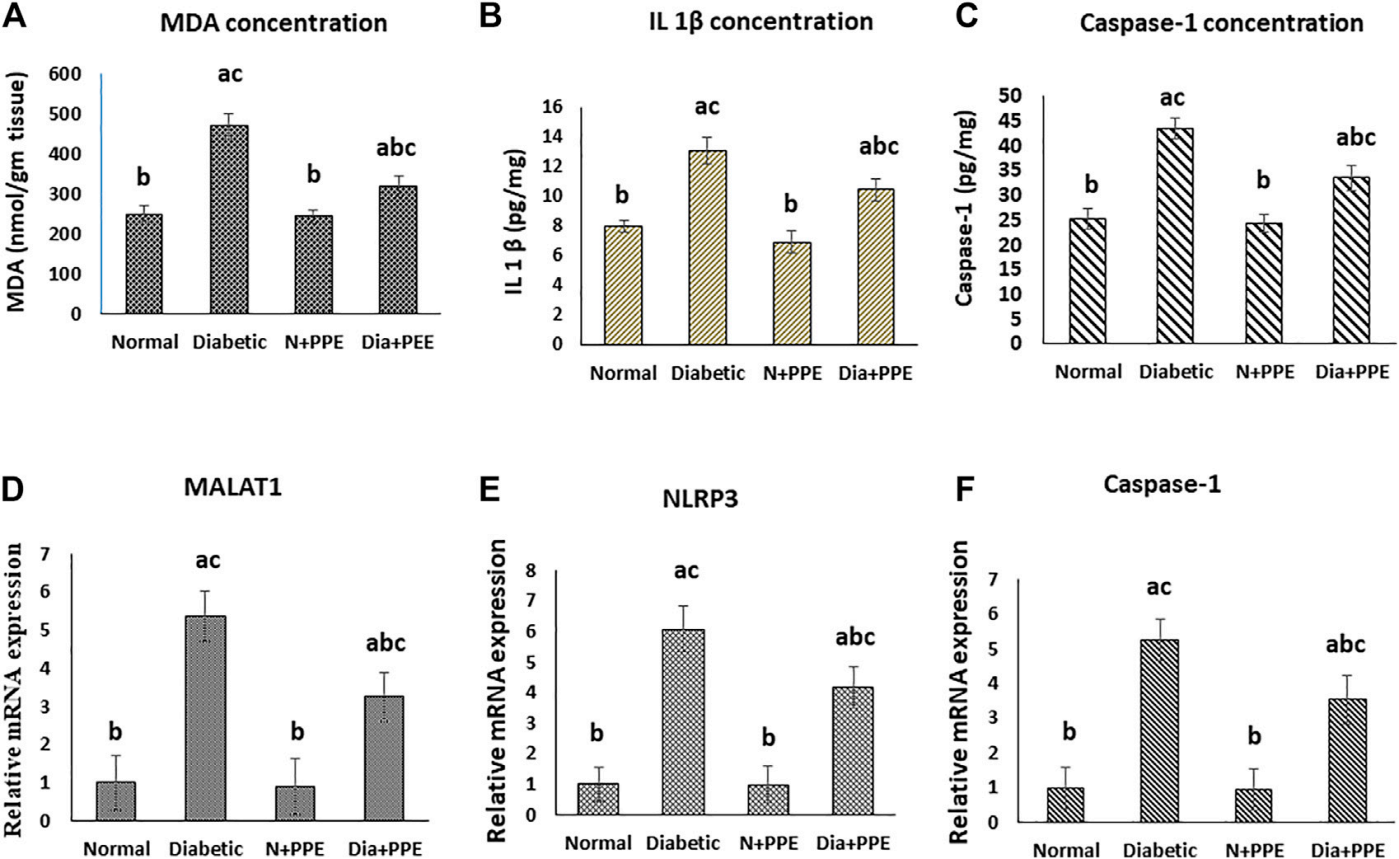
Effect of the pomegranate peel extract treatment on heart tissue of the studied rat groups. **(A)**: MDA level as marker of lipid peroxidation, **(B)**: IL-1β content detected by ELISA. **(C–E)**: gene expression analysis of lncRNA-MALAT1, NLRP3, and caspase-1 respectively. The gene expression was measured using quantitative RT-PCR. Data are expressed as the mean ± SD. n = 7 for MDA and IL-1β concentration while for gene expression analysis n = 3. IL-1β: interleukin 1 beta; MALAT1: Metastasis associated lung adenocarcinoma transcript 1; MDA: malondialdehyde; NLRP3: nucleotide-binding domain (NOD)-like receptor protein 3, Normal: normal rats (negative control group), Diabetic: non-treated diabetic rats (positive control group), N+PPE: normal rats treated with the pomegranate peel extract, Dia+PPE: diabetic rats treated with the pomegranate peel extract.

Malondialdehyde (MDA) concentration was determined in the cardiac tissue as a marker of myocardial lipid peroxidation. The concentration of MDA was significantly higher in the cardiac tissue of the diabetic rats in the positive control group (470.05 ± 32.21 nmol/g tissue) than in non-diabetic rats in the negative control group (253.13 ± 34.92 nmol/g tissue) (*p* < 0.001). More interestingly, prophylactic treatment of the diabetic rats with the PPE significantly decreased MDA levels in the myocardium tissue (322.11 ± 24.31 nmol/g tissue) compared to the untreated diabetic rats in the positive control group (*p* < 0.001). MDA concentration in the heart of the normal rats treated with the PPE (244.27 ± 27.83 nmol/g tissue) showed no significant difference from MDA concentration in the heart of the rats in the negative control group (Figure 4A).

#### 3.2.5 Effect on the level of IL-1β and caspase-1 in the myocardial tissue

The concentrations of the pro-inflammatory cytokine **(**IL-1β), which was released as a result of the NLRP3 activation, and caspase-1 (IL-1-converting enzyme) were determined in the cardiac tissues of the studied rat groups as a marker of pyroptosis. The concentrations of IL-1β and caspase-1 were significantly higher in the cardiac tissue of the diabetic rats in the positive control group (13.11 ± 0. 88 pg/mg and 43.49 ± 2.12 pg/mg, respectively) than in the non-diabetic rats in the negative control group (7.67 ± 0. 46 pg/mg and 25.28 ± 2.23, respectively) (*p* < 0.001). Prophylactic treatment of the diabetic rats with the PPE significantly decreased IL-1β and caspase-1 concentrations in the myocardium tissue (10.44 ± 0.76 pg/mg and 33.55 ± 2.52 pg/mg, respectively) compared to the untreated diabetic rats in the positive control group (*p* < 0.001). Moreover, the concentrations of IL-1β and caspase-1 in the heart of the normal rats treated with the PPE (6.96 ± 0.75 pg/mg and 24.44 ± 1.923, respectively) showed no significant difference from their concentrations in the heart of the normal rats in the negative control group (Figures 4B, C).

#### 3.2.6 Effect on lncRNA-MALAT1 and pyroptosis in the cardiac tissue

Our RT-PCR data showed that the gene expressions of lncRNA-MALAT1 and the pyroptosis markers (NLRP3 and caspase-1) in the cardiac tissue of the diabetic rats in the positive control group (5.374 ± 0.715, 6.079 ± 0.742, and 5.248 ± 0.584 folds, respectively) were significantly higher than their gene expressions in the cardiac tissue of the non-diabetic rats in the negative control group (1-fold, *p* < 0.001). Meanwhile, prophylactic administration of the PPE induced significant a decrease in the gene expressions of lncRNA-MALAT1 and pyroptosis markers (NLRP3 and caspase-1) in the cardiac tissue of the diabetic rats (3.252 ± 0.643, 4.162 ± 0.668, and 3.534 ± 0.745 fold, respectively, *p* < 0.05). Our results showed that the gene expressions of lncRNA-MALAT1 and the pyroptosis markers (NLRP3 and caspase-1) in the cardiac tissue of the non-diabetic rats treated with the PPE (0.913 ± 0.742, 0.974 ± 0.632, and 0.941 ± 0.553 fold, respectively) showed no significant difference from their gene expressions in the cardiac tissue of the non-diabetic rats in the negative control group (1-fold) (Figures 4D–F).

#### 3.2.7 Histopathological examinations

**FIGURE 5:**
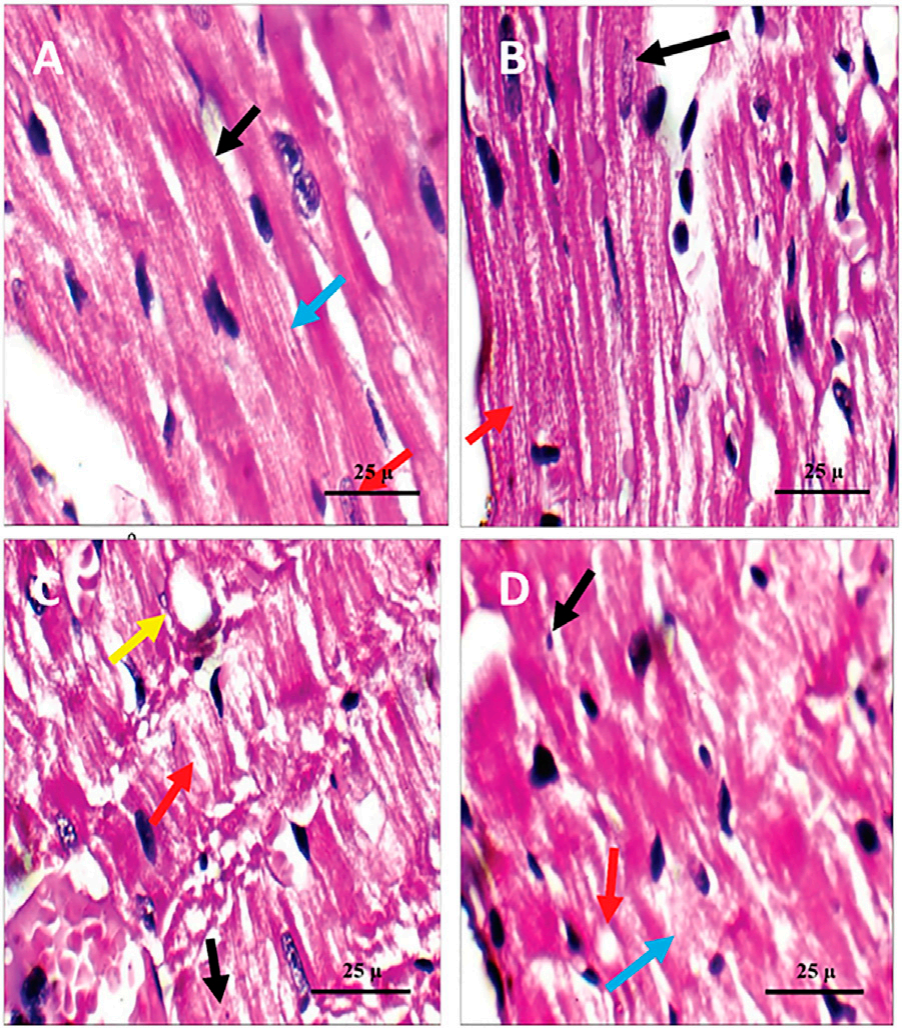
Microscopic pictures of the cardiac tissue of the studied rat groups stained with H&E. **(A)** Cardiac wall of the non-diabetic rats in the negative control group showing viable cardiac muscle fibers with distinct cell borders (black arrow), preserved cross striations (blue arrow), and central oval\elongated nuclei (red arrow). **(B)** Cardiac wall of the non-diabetic rats treated with the pomegranate peel extract showing cardiac muscle fibers with centrally located nuclei (black arrow) and preserved cross striations (red arrow). **(C)** Cardiac wall of the diabetic rats in the positive control group showing scattered cardiac muscle fibers with small pyknotic nuclei (black arrow), partial loss of cross striations (red arrow), mildly congested intervening blood capillaries (blue arrow), and scattered cytoplasmic vacuoles (yellow arrow). **(D)** Cardiac wall of the diabetic rats treated with the pomegranate peel extract showing mildly scattered cardiac muscle fibers with small pyknotic nuclei (black arrow), few scattered cytoplasmic vacuoles (blue arrow), and partial loss of cross striations (red arrow).

Histopathological examination revealed that the cardiac wall of the non-diabetic rats in the negative control group showed normal viable cardiac muscle fibers with distinct cell borders, preserved cross striations, and central oval\elongated nuclei (Figure 5A). The cardiac wall of the non-diabetic rats treated with the PPE showed an appearance similar to that of the normal control group as the cardiac muscle fibers showed centrally located nuclei and preserved cross striations (Figure 5B). Meanwhile, the cardiac wall from the diabetic rats in the positive control group showed scattered cardiac muscle fibers with small pyknotic nuclei, partial loss of cross striations, mildly congested intervening blood capillaries, and scattered cytoplasmic vacuoles (Figure 5C). More interestingly, the cardiac wall of the diabetic rats treated with the PPE showed mildly scattered cardiac muscle fibers with small pyknotic nuclei, few scattered cytoplasmic vacuoles, and partial loss of cross striations (Table 2; Figure 5D).

**TABLE 2:** Effect of pomegranate peel extract (PPE) on histopathological scoring of alterations caused by diabetes in rats’ cardiac tissues of the studied groups.

Group	Normal	Diabetic	N + PPE	Dia + PPE
Scattered cardiac muscle fibers	−	**++**	−	**+**
Pyknotic nuclei	−	**+**	−	**+**
Edema	**−**	**++**	−	**−**
Cytoplasmic vacuoles	−	++	−	+
Congestive blood vessel	−	+	−	−

Normal: normal rats (negative control group); diabetic: non-treated diabetic rats (positive control group); N + PPE: normal rats treated with the PPE; Dia + PPE: diabetic rats treated with the PPE.

#### 3.2.8 Effect on TGF-β as a marker for fibrosis

**FIGURE 6:**
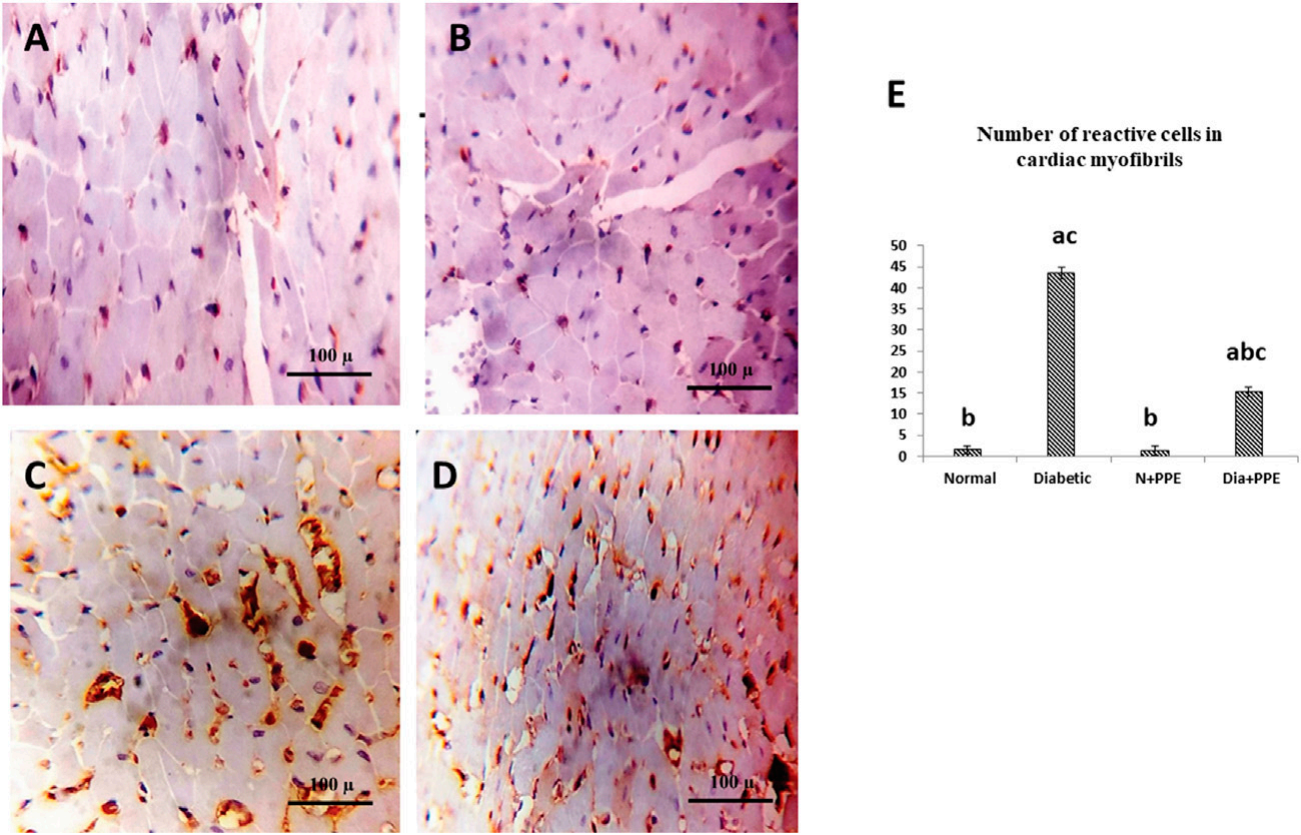
Immunostained cardiac myofibrils against TGF-β. **(A)** Non-diabetic rats in the negative control group cardiac wall showing negative cell membrane reactivity in cardiac myofibrils. **(B)** Cardiac wall of non-diabetic rats treated with the pomegranate peel extract showing negative cell membrane reactivity in cardiac myofibrils. **(C)** Cardiac wall of the diabetic rats in the positive control group showing marked cell membrane reactivity in cardiac myofibrils. **(D)** Cardiac wall of the diabetic rats treated with the pomegranate peel extract showing weak cell membrane reactivity in cardiac myofibrils. **(E)** Illustrative figure demonstrating the average number of reactive cells in cardiac myofibrils against TGF-β of the studied rat groups. Data are presented as mean ± SD; *n* = 6. Normal: normal rats (negative control group). Diabetic: non-treated diabetic rats (positive control group). N + PPE: normal rats treated with the PPE. Dia + PPE: diabetic rats treated with the PPE. a: significant *versus* negative control group; b: significant *versus* positive control group, c: significant *versus* treated normal group; *p* < 0.05.

To confirm fibrosis in the cardiac tissue, immunostaining of TGF-β was performed. Immunostained cardiac myofibrils against TGF-β showed a cardiac wall with negative cell membrane reactivity in the non-diabetic rats in the negative control group and the non-diabetic rats treated with the PPE (Figures 6A, B). Meanwhile, cardiac myofibrils from the diabetic rats in the positive control group showed marked cell membrane reactivity in the cardiac wall (Figure 6C). More interestingly, the cardiac myofibrils from diabetic rats treated with the PPE showed a cardiac wall with weak cell membrane reactivity (Figure 6D). Figure 6E illustrates that the number of reactive cells against TGF-β in cardiac myofibrils from diabetic rats treated with PPE was significantly lower than their number in diabetic untreated rats (*p* < 0.001).

#### 3.2.9 Determination of the amount of collagen in the cardiac tissue of diabetic rats

**FIGURE 7:**
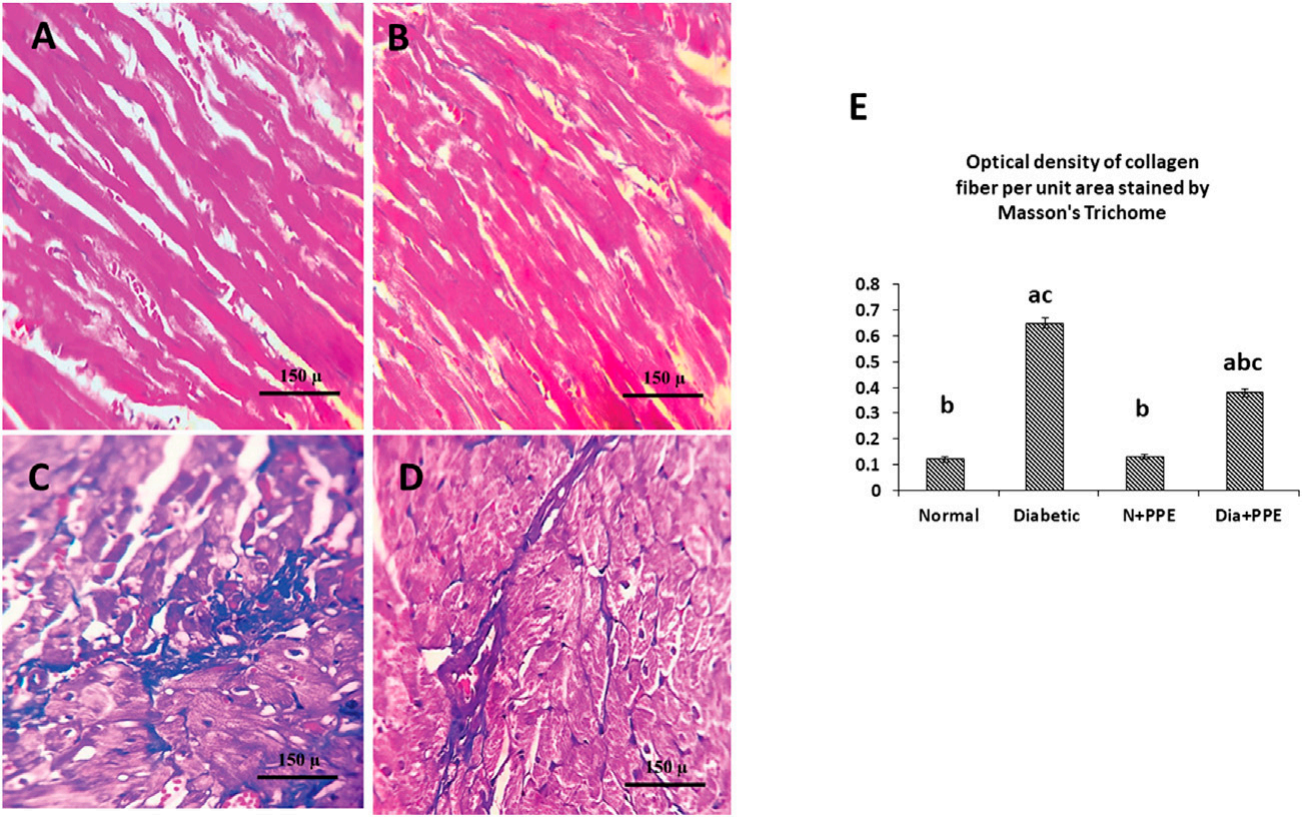
Masson’s trichome staining of the cardiac tissues of the studied rat groups. **(A)** Cardiac tissue from the non-diabetic rats in the negative control group showing normal delicate fibrous strand. **(B)** Cardiac tissue from the non-diabetic rats treated with the pomegranate peel extract showing an appearance similar to that of interstitial collagen as the negative control group. **(C)** Cardiac tissue from the diabetic rats in the positive control group showing thick fibrous bands. **(D)** Cardiac tissue from the diabetic rats treated with pomegranate peel extract group illustrating a mild amount of collagen fibers. **(E)** Illustrative figure demonstrating the quantitative measurement of the optical density of collagen fiber per unit area stained by Masson’s trichome. Data are presented as mean ± SD; *n* = 6. Normal: normal rats (negative control group). Diabetic: non-treated diabetic rats (positive control group). N + PPE: normal rats treated with the PPE. Dia + PPE: diabetic rats treated with the PPE. a: significant *versus* negative control group; b: significant *versus* positive control group, c: significant *versus* treated normal group; *p* < 0.05.

For collagen amount determination in the rat myocardium, Masson staining was used. Microscopic pictures of Masson’s trichome staining of the cardiac tissues of the non-diabetic rats in the negative control group and non-diabetic rats treated with the PPE showed normal delicate fibrous strands (Figures 7A, B). Meanwhile, the cardiac tissues of the diabetic rats in the positive control group showed thick fibrous bands (Figure 7C). In addition, the cardiac tissues of the diabetic rats treated with the PPE illustrated a mild amount of collagen fibers (Figure 7D).

Figure 7E illustrates the quantitative measurements of the optical density of collagen fibers in the heart of the studied rat groups using the Masson trichrome technique. The amount of collagen fiber per unit area in cardiac myofibrils from diabetic rats was significantly higher than its amount in the non-diabetic rats in negative control group (*p* < 0.001). Moreover, prophylactic treatment of the diabetic rats with PPE significantly decreased the amount of collagen fiber in cardiac myofibrils per unit area (*p* < 0.001).

## 4 Discussion

Diabetic cardiomyopathy is a recognized complication of diabetes mellitus, with a prevalence of 1.1% (Dandamudi et al., 2014). Oxidative stress and lipid peroxidation are core factors in the pathophysiology of diabetes-induced cardiac injury (El-Missiry et al., 2015). These factors are a consequence of higher reactive oxygen species generation produced by metabolic disturbances. The disease is characterized by myocardial hypertrophy, myocardial fibrosis, and an elevated level of cTn-1. Currently, there is no specific treatment for DC, and the cardioprotective strategies for DC prevention include the use of antioxidants, antifibrotic agents, and anti-inflammatory agents (Lorenzo-Almorós et al., 2022). Our findings showed, for the first time, that PPE ameliorates the impact of diabetes on the myocardium and revealed the likely mechanisms of its favorable effects, which are downregulation of lncRNA-MALAT1, with subsequent downregulation of the pyroptosis-related genes (NLRP3 and caspase-1), and reduction of IL-1β. This study revealed that PPE treatment increased the survival rate and protected against the development of cardiac hypertrophy in diabetic rats. Our results showed that the survival rate of the rats in the positive control group was 53.33%, while the survival rate of the rats in the diabetic treated group was 73.33%. Moreover, prophylactic treatment of the diabetic rats with PPE significantly decreased the relative heart weight compared to the untreated diabetic rats in the positive control group (*p* < 0.001).

Diabetes mellitus notoriously leads to a myriad of complications, such as DC that may be eventually complicated by the occurrence of heart failure and arrhythmia. Metabolic derangement, mitochondrial dysfunction, autophagy, and inflammatory cell death are all implicated in initial myocardial hypertrophy and, eventually, fibrosis (Yan et al., 2020; Prandi et al., 2022).

The animal model used in this study is of type 1 diabetes mellitus. In patients with T1DM, the disease is characterized by hyperglycemia and dyslipidemia as frequently occurring metabolic abnormalities. Both are associated with an increased cardiovascular risk (Zabeen et al., 2018; Jebraeili et al., 2021). Our results showed that prophylactic treatment of the diabetic rats with PPE significantly improved the serum lipid profile (*p* < 0.05), with no significant effect on the blood glucose concentration.

Our results were in agreement with those of Hou et al. (2019), who reported that pomegranate extract regulates lipid metabolism and improves lipid profile in metabolic-disorder-associated diseases such as non-alcoholic fatty liver disease and type 2 diabetes. On the other hand, our findings showed that PPE could improve the pathological effect of diabetes on the lipid profile of rats, but it did not significantly affect the normal lipid profile in the non-diabetic rats.

Serum cTn1 is a common marker of myocardial injury (El-Mansi and Al-Kahtani, 2019; Jin et al., 2021). This study showed that prophylactic treatment of the diabetic rats with PPE significantly decreased the serum cTn1 concentration compared to the untreated diabetic rats in the positive control group (*p* < 0.001). Our results were in line with those of Jin et al. (2021), who found that increased serum cTn1 in diabetic mother rats was decreased by prophylactic treatment with PPE.

In diabetes, superoxide anion radicals are overproduced in the mitochondria, subsequently inducing an increased polyol pathway flux and activation of the receptor for AGE and protein kinase C isoforms. It is of note that such pathways not only directly cause diabetic cardiomyopathy but are also leading sources of ROS (Prandi et al., 2022). The ROS and oxidative stress lead to cellular malfunction injury through several mechanisms, including protein oxidation, lipid peroxidation, DNA damage, and oxidative changes in microRNAs (Pizzino et al., 2017).

In this study, we determined MDA in the cardiac tissue as a marker of lipid peroxidation. MDA is an end product of the oxidation of polyunsaturated fatty acids containing more than two double bonds and its increased level reflects high levels of ROS and oxidative stress. Our data showed that the prophylactic treatment of the diabetic rats with PPE significantly decreased the MDA levels in the myocardium tissue compared to the untreated diabetic rats in the positive control group (*p* < 0.001). Our findings agreed with those of Jin et al. (2021), who found that the increased MDA in the myocardium of diabetic mother rats was decreased by prophylactic treatment with allisartan isoproxil. In the current study, the PPE could decrease the elevated pathological level of MDA due to hyperglycemia in diabetic rats, but it did not significantly affect the normal MDA level in non-diabetic rats. This effect could be attributed to the antioxidant and ameliorative action of pomegranate polyphenolic constituents, which scavenge the free radicals through the AMPK-Nrf2 signaling pathway and thus decrease lipid peroxidation (Aboonabi et al., 2014; Sun et al., 2016; Akuru et al., 2022).

Pyroptosis is an inflammatory programmed cell death which is strongly associated with the development of DC. ROS and hyperglycemia associated with diabetes activate NLRP3 oligomerization which triggers the activation of caspase-1. Active caspase-1 converts the inactive pro-inflammatory cytokine IL-1β into its active form and facilitates the release of active IL-1β out of the cell (Xue et al., 2019; Zheng and Li, 2020). Our real-time PCR data showed that the prophylactic treatment with PPE significantly decreased the gene expression of pyroptosis markers (NLRP3 and caspase-1) in the cardiac tissue of the diabetic rats compared to those of rats in the positive control group (*p* < 0.05). This effect could be explained by the antioxidant effect of PPE, thus deactivating NLRP3 and its related processes.

In the present study, the ELISA technique was performed for further confirmation of our real-time PCR findings. The concentrations of caspase-1 and IL-1β in the cardiac tissues of the studied rat groups were determined by ELISA. The prophylactic treatment of the diabetic rats with PPE significantly decreased the concentration of caspase-1 and IL-1β in the myocardium tissue compared to the untreated diabetic rats in the positive control group (*p* < 0.001). In the present study, the PPE could inhibit the diabetes-activated pyroptosis pathway in diabetic rats, but it did not significantly affect the pyroptosis in non-diabetic rats as it is un-activated and has normal glucose level and no or low level of ROS.

The lncRNA-MALAT1 is involved in several pathophysiological mechanisms in multiple diseases, including DC (Zhang et al., 2016; Abdulle et al., 2019). LncRNA-MALAT1 is upregulated in DC, and the knockdown of MALAT1 protects against the development of DC. Intriguingly, MALAT1 could be a novel therapeutic target for DC (Shi et al., 2021). Gene expression analysis in this study showed that prophylactic treatment with PPE significantly decreased the gene expression of lncRNA-MALAT1 in the cardiac tissue of the diabetic rats compared to those of rats in the positive control group (*p* < 0.05). On the other hand, PPE had no significant effect on the normal level of lncRNA-MALAT1 in non-diabetic rats.

Our findings revealed, for the first time, that the protective effect of PPE on DC could be due to the inhibition of pyroptosis, specifically by downregulating lncRNA-MALAT1. Our findings were supported by Wu et al. (2021), who found that lncRNA-MALAT1 promotes pyroptosis induced by high glucose concentration in the H9C2 cardiomyocyte via downregulating miR-141-3p.

Transforming growth factor-β is the master cytokine that mediates fibrosis in various tissues as a consequence of tissue injury and inflammation (Khalil et al., 2017). In the current study, immunostaining of TGF-β was performed to confirm fibrosis in the cardiac tissue. Moreover, Masson staining was used for the determination of the collagen amount. The microscopic examination of the cardiac tissues of the studied rat groups revealed that prophylactic treatment with PPE significantly decreased the number of TGF-β-positive cells and the amount of collagen in the cardiac tissue of the diabetic rats. The current data were in line with the findings of Dab et al. (2022), who reported that PPE modulates the cardiac extracellular matrix and decreases the content of hydroxyproline and total collagen in a rat diabetic model.

Metabolic derangement, mitochondrial dysfunction, and inflammatory cell death are all implicated in myocardial hypertrophy and, eventually, fibrosis (Yan et al., 2020; Prandi et al., 2022). Fibrosis is a key feature of DC, leading to increased stiffness and loss of contractile function in diastolic and systolic DC types, respectively. Such pathological processes lead to complications, including heart failure and arrhythmias (Russo and Frangogiannis, 2016; Youssef et al., 2022).

Our histopathologic and immunohistochemistry findings were in agreement with our biochemical data. The histopathologic and immunohistochemistry staining showed that prophylactic treatment with PPE prevented deleterious histopathological alterations in the cardiac myocytes and ameliorated the tissue damage caused by diabetes. The favorable changes were indicated by the attenuated collagen accumulation and the decreased number of TGF-β-positive cells.

Our current findings have impactful translational meaning in clinical practice. Applied to all types of diabetes mellitus, DC may be diagnosed in patients with diabetes as any cardiac systolic, or at least, moderate diastolic dysfunction, with no history of other cardiac diseases including hypertension, coronary, and significant valvular or congenital heart disease (Dandamudi et al., 2014). A prevalence study by Dandamudi et al., 2014 revealed that DC occurs in 1.1% of the general population. About 17% of diabetic patients included in their study had DC (54.4% with diastolic dysfunction). Out of the studied diabetic patients, 22% developed heart failure within the 9-year follow-up period (Dandamudi et al., 2014).

The prevalence of diabetes mellitus type 2 is more than 20-fold higher than that of type 1; therefore, diabetic cardiomyopathy is mainly observed in type 2 diabetic patients. However, our model represents the two main pathological derangements linking DM, in all its types, to DC, which are hyperglycemia and dyslipidemia.

The PPE is rich in ellagitannins and anthocyanins, which are strong antioxidant agents. In our study, punicalagin, pedunculagin, valoneic acid dilactone, HHDPG, and ellagic acid are the ellagitannins detected, whereas cyanidin-3-*O*-glucoside and delphinidin-3-*O*-glucoside were the major anthocyanins. Ellagitannins are metabolized to ellagic acid and then to urolithin compounds, which are considered responsible for the biological activities of ellagitannins (Villalba et al., 2019). Punicalagin demonstrated a cardioprotective effect in diabetic conditions where it significantly decreased elevated cTn-1 to normal levels, attenuated IL-1b, IL-6, and TNF-α, and ameliorated the lipid profile in a streptozotocin-induced diabetic model (El-Missiry et al., 2015). Urolithin compounds A and B prevent the development of diabetic cardiomyopathy (Savi et al., 2017; Chen et al., 2022; Selma et al., 2021) by increasing the expression of sarco(endo)plasmic reticulum calcium ATPase 2 (SERCA2) and the activation of SIRT1; consequently, glycolysis is increased *via* a positive modulation of pyruvate dehydrogenase activity and ameliorated cardiac function. The anti-inflammatory effect of urolithin B was evidenced by a decrease in some inflammatory cytokines, including interleukin-6 (IL-6), interferon-γ (IFN-γ), tumor necrosis factor-α (TNF-α), interleukin-4 (IL-4), and IL-1β through the inhibition of nuclear factor-κ-gene binding (NF-κB) activation and mitogen-activated protein kinase (MAPK). In addition, urolithin B exhibits antioxidant activity mediated by decreasing the production of ROS and the expression of NADPH oxidase subunit in addition to upregulating heme oxygenase-1 expression via the Nrf2/ARE signaling pathway (Lee et al., 2019).

It was reported that ellagic acid stimulates cardiac silent information regulator 1 signaling, thus protecting against DC in rats (Altamimi et al., 2020). Ellagic acid lowers triglyceride content, malondialdehyde level, (IL)-beta, IL-6, and TNF-alpha in the cardiac tissues (Chao et al., 2009). Additionally, ellagic acid upregulates cardiac mRNA expression of glutathione peroxidase, superoxide dismutase, and catalase, which accounts for its antioxidant effect (Chao et al., 2009).

Anthocyanins were reported to have a cardioprotective effect against streptozotocin-associated DC (Chen et al., 2016). Anthocyanins upregulate Nrf2, thus inducing the production of endogenous antioxidants (Sapian et al., 2022). Moreover, anthocyanins are evidently effective against increased cytokine production, lipid peroxidation, and inflammation. Cyanidin-3-*O*-glucoside and delphinidin-3-*O*-glucoside, detected in our study, are among the anthocyanins that have positive effects on diabetes-associated complications (Chen et al., 2016). Cyanidin-3-*O*-glucoside and delphinidin-3-*O*-glucoside showed cardioprotective action in DC due to their antioxidant properties (Liobikas et al., 2016; Li et al., 2018).

Nutritional intervention has become an essential component of the management plans for chronic diseases. Thus, initiatives and campaigns planned to increase vegetable and fruit intake are required and justified. The promotion of vegetable and fruit consumption by health policies can be a promising strategy to help prevent and improve the outcome of diabetes mellitus, among other chronic illnesses (Boeing et al., 2012). Guidance may include specific research-based advice, e.g., promoting PPE intake in diabetic patients to hopefully prevent and improve the outcome of DC.

## 5 Conclusion

The current study demonstrated that pomegranate peel extract showed a cardioprotective effect in diabetic rats, most likely due to its unique antioxidant, anti-inflammatory, and antifibrotic properties and its ability to improve the lipid profile. The protective effect of PPE could be due to the inhibition of the NLRP3/caspase-1/IL1β signaling pathway and downregulation of lncRNA-MALAT1. Thus, PPE could be a promising protective remedy against the development of DC. Clinical studies are recommended to evaluate the effect of PPE as a cardioprotective supplement in DC.

## Data Availability

The original contributions presented in the study are included in the article/Supplementary Material; further inquiries can be directed to the corresponding authors.
